# BzATP Activates Satellite Glial Cells and Increases the Excitability of Dorsal Root Ganglia Neurons In Vivo

**DOI:** 10.3390/cells11152280

**Published:** 2022-07-23

**Authors:** Zhiyong Chen, Chi Zhang, Xiaodan Song, Xiang Cui, Jing Liu, Neil C. Ford, Shaoqiu He, Guangwu Zhu, Xinzhong Dong, Menachem Hanani, Yun Guan

**Affiliations:** 1 Department of Anesthesiology and Critical Care Medicine, Johns Hopkins University School of Medicine, Baltimore, MD 21205, USA; 102584@hrbmu.edu.cn (Z.C.); czhan110@jhmi.edu (C.Z.); danxsong@163.com (X.S.); xcui10@jhu.edu (X.C.); jliu307@jhu.edu (J.L.); nford9@jhmi.edu (N.C.F.); she11@jhmi.edu (S.H.); gzhu10@jhmi.edu (G.Z.); 2 The Solomon H. Snyder Department of Neuroscience, Center for Sensory Biology, Johns Hopkins University School of Medicine, Baltimore, MD 21205, USA; xdong2@jhmi.edu; 3 Howard Hughes Medical Institute, Johns Hopkins University School of Medicine, Baltimore, MD 21205, USA; 4 Laboratory of Experimental Surgery, Hadassah-Hebrew University Medical Center, Faculty of Medicine, Hebrew University of Jerusalem, Mount Scopus, Jerusalem 91240, Israel; hananim@mail.huji.ac.il; 5 Department of Neurological Surgery, Johns Hopkins University School of Medicine, Baltimore, MD 21205, USA

**Keywords:** satellite glial cell, dorsal root ganglion, purinergic receptor, calcium imaging, pain, mice

## Abstract

The purinergic system plays an important role in pain transmission. Recent studies have suggested that activation of P2-purinergic receptors (P2Rs) may be involved in neuron-satellite glial cell (SGC) interactions in the dorsal root ganglia (DRG), but the details remain unclear. In DRG, P2X7R is selectively expressed in SGCs, which closely surround neurons, and is highly sensitive to 3’-O-(4-Benzoyl) benzoyl-ATP (BzATP). Using calcium imaging in intact mice to survey a large number of DRG neurons and SGCs, we examined how intra-ganglionic purinergic signaling initiated by BzATP affects neuronal activities in vivo. We developed *GFAP*-GCaMP6s and *Pirt*-GCaMP6s mice to express the genetically encoded calcium indicator GGCaM6s in SGCs and DRG neurons, respectively. The application of BzATP to the ganglion induced concentration-dependent activation of SGCs in *GFAP*-GCaMP6s mice. In *Pirt*-GCaMP6s mice, BzATP initially activated more large-size neurons than small-size ones. Both glial and neuronal responses to BzATP were blocked by A438079, a P2X7R-selective antagonist. Moreover, blockers to pannexin1 channels (probenecid) and P2X3R (A317491) also reduced the actions of BzATP, suggesting that P2X7R stimulation may induce the opening of pannexin1 channels, leading to paracrine ATP release, which could further excite neurons by acting on P2X3Rs. Importantly, BzATP increased the responses of small-size DRG neurons and wide-dynamic range spinal neurons to subsequent peripheral stimuli. Our findings suggest that intra-ganglionic purinergic signaling initiated by P2X7R activation could trigger SGC-neuron interaction in vivo and increase DRG neuron excitability.

## 1. Introduction

In the dorsal root ganglion (DRG), the neuron and the surrounding satellite glial cells (SGCs) form a distinct morphological “neuron-glial unit” [1,2,3]. In this complex, SGCs form a basket-like structure that enwraps the neuron, with a narrow space of ~20 nm separating the neurons and SGCs [2]. This unique layout enables SGCs to closely monitor neuronal functions and to communicate with the neurons through both diffusible (e.g., paracrine release of glial modulators) and non-diffusive mechanisms (e.g., gap junctions) [1,3].

Within the ganglion, neurons may release adenosine 5’-triphosphate (ATP) [4], an endogenous ligand to P2 receptors (P2R), from their somata after the opening of L-type channels. On the other hand, ATP and other glial transmitters such as glutamate and cytokines can also be released from SGCs [1,4,5,6]. Previous studies have suggested that purinergic signaling participates in the bi-directional communication between neurons and SGCs, and released ATP may have important functional consequences on neuronal excitability in vitro [4,7,8,9]. However, the ways in which intra-ganglionic purinergic signaling modulates SGC and DRG neuronal activity in vivo, as well as how it may affect nociceptive transmission, remain unclear.

To answer these important questions, we recently took an in vivo calcium imaging approach and examined intra-ganglionic purinergic signaling through monitoring activities of hundreds of DRG neurons and SGCs in intact mice [10]. SGCs are the major type of glial cells in DRG [1]. They are electrically inexcitable due to the absence of voltage-gated sodium and calcium channels [1,11]. Cytosolic calcium responses in both SGCs and neurons can be measured by calcium imaging, which represents an important approach for investigating calcium-based excitability [1,12]. We expressed genetically encoded calcium indicator GCaMP6s in DRG neurons of *Pirt*-GCaMP6s mice and also developed *GFAP*-GCaMP6s mice to express GCaMP6s in SGCs and astrocytes [10]. In sensory ganglia, P2X3Rs are predominantly expressed in small-size neurons, and P2X7Rs are mostly expressed in SGCs [13,14,15,16]. Our previous findings showed that ganglionic application of α,β-methyleneadenosine 5-triphosphate (α,β-MeATP), which activates neuronal P2X2/3R, induces robust activation of small-size DRG neurons, followed by delayed large-size neurons activation [10].

Currently, there is a lack of information on how the activation of glial P2Rs may affect DRG neuronal excitability in vivo. In the DRG, P2X7Rs are mostly expressed in SGCs but not in neurons [4,7,15]. Previous studies of SGC-neuron interaction were conducted mainly in vitro [3,4,9,17,18,19] by using a co-culture system and non-selective P2R agonists (e.g., ATP), making it difficult to ascertain the specific receptor mechanisms involved. Unlike P2X3Rs, P2X7Rs expressed in SGCs are relatively insensitive to ATP and α,β-MeATP [5,20,21,22]. In contrast, BzATP is a potent P2X7R-preferred agonist, but only weakly interacts with other purinergic receptors [8,15,16,20,22]. Here, we used it as a pharmacological tool to preferentially initiate purinergic signaling in SGCs, and examined how it subsequently affects the excitability of DRG neurons and nociceptive transmission in vivo. We hypothesized that BzATP will excite SGCs, trigger DRG neuron activation via SGC–neuron interaction, and sensitize DRG neurons to subsequent peripheral stimulation and thus facilitate nociceptive transmission.

## 2. Materials and Methods

### 2.1. Animals

Adult *Pirt*-GCaMP6s mice and *GFAP*-GCaMP6s mice (25–35 g, both sexes) were used for in vivo calcium imaging of neurons and SGCs, respectively. *Pirt*-GCaMP6s mice were generated by crossing *Pirt*-Cre mice with *Rosa26-loxP-STOP-loxP-GCaMP6s* mice, as described in previous studies [23,24,25]. Since *Cre* recombinase is controlled by the *Pirt* promoter and *Pirt* is expressed only in DRG neurons but not in glial cells or the central nervous system [26], GCaMP6s is expressed specifically in most DRG neurons. *GFAP* promoter is expressed primarily in SGCs in the DRG [11], and *GFAP*-GCaMP6s mice were generated by crossing *GFAP*-Cre mice with *Rosa26-loxP-STOP-loxP-GCaMP6s* mice. We also crossed *Pirt*-GCaMP6s mice and *GFAP*-GCaMP6s mice to generate *Pirt:GFAP*-GCaMP6s mice, which express GCaMP6s in both neurons and SGCs.

*GFAP*-Cre mice were purchased from Jackson Laboratory (Bar Harbor, ME, B6.Cg-Tg(Gfap-cre)77.6Mvs/2J, Stock No: 024098), and *Rosa26-loxP-STOP-loxP-GCaMP6s* mice were received as a gift from Dr. Dwight E. Bergles in the Solomon H. Snyder Department of Neuroscience, School of Medicine, Johns Hopkins University (Baltimore, MD, USA). All transgenic mice were backcrossed to C57BL/6J mice for at least 10 generations. Adult wild-type C57BL/6J mice (25–35 g, both sexes, Jackson Laboratory) were used for the electrophysiologic recording of dorsal horn neurons. Mice were housed in groups of three to five on a standard 12 h light/12 h dark cycle with free access to food and water. All animal work was approved by the Animal Care and Use Committee of Johns Hopkins University and complied with the National Institutes of Health Guide for the Care and Use of Laboratory Animals to ensure minimal animal use and discomfort.

### 2.2. In Vivo Calcium Imaging

We conducted imaging of L4 DRG neurons, the axons of which are included in the sciatic nerve and innervate the hindpaw, leg, and thigh in mice. L4 DRG was chosen because of the easier surgical procedure to expose this ganglion and because it has been the most studied in previous in vivo calcium-imaging studies [10,23,24,25] and hence allows comparisons between different studies. Mice were anesthetized with 2% isoflurane. After the lower lumbar spine (L3–L5 vertebra bones) was exposed, the L4 DRG transverse processes were cleaned and the dorsal aspect near the vertebra was removed to expose the underlying ganglion, without damaging the DRG, nerve roots, or spinal cord. After removing the epineurium, the L4 DRG was bathed in a pool of extracellular artificial cerebrospinal fluid (ACSF, ~1 mL), which contained 120 mM NaCl, 3 mM KCl, 1.1 mM CaCl_2_, 10 mM glucose, 0.6 mM NaH_2_PO_4_, 0.8 mM MgSO_4_, and 18 mM NaHCO_3_. L4 DRG was placed under a 25 ×/0.95 W VISIR 0.17 long-working distance (2.5 mm) water immersion objective, and changes in fluorescence intensity in neurons and SGCs were imaged by laser-scanning confocal microscopy (Leica TCS SP8, Wetzlar, Germany). Time-lapse z-stacks (frames) of the intact DRG were acquired at 10 s/frame and 512 × 512 pixel resolution. Each frame consisted of 10 scans (one scan/second), and 26 frames were acquired for scanning each DRG (laser wavelength: 488 nm, laser power: 6%, scan speed: 400 Hz).

In concentration-response studies, increasing concentrations of BzATP (5, 50, and 500 µM) were applied at the ganglion, with washout after each dose. High-concentration drug solution (0.1 mL) was added into the bath (~1 mL ACSF) as one bolus with a pipette to reach the final working concentration. After imaging was complete for each dose, the drug was washed out with ACSF, followed by an interval of 30 min before testing the next higher dose. This interval allows a sufficient recovery from the previous drug action and a complete return of cytosolic fluorescence to the pre-drug baseline. The body temperature of the mice was maintained with a heating pad at 36.0–37.0 °C, as monitored by a rectal probe.

### 2.3. Quantification of Calcium Imaging

Cytosolic calcium responses were indicated as the increase in green fluorescence intensity. The raw images (Tagged Image File Format) were exported and analyzed using image analysis software (ImageJ, National Institutes of Health, Bethesda, MD, USA https://imagej.nih.gov/ij/ accessed on 2 May 2022) [27,28] and LIF (Leica Microsystems GmbH). An experimenter traced the activated cells manually to determine cell size and fluorescence intensity. Fluorescence intensity at the baseline level was taken as F_0_. Evoked calcium response was expressed as the ratio of the post-treatment fluorescence intensity (F) to the basal level, as shown in our previous studies [10,25]. Activation in neurons was defined as an increase in F/F_0_ ≥ 1.5 fold.

### 2.4. In Vivo Extracellular Recording of Spinal Wide-Dynamic Range (WDR) Neurons

Electrophysiological recordings were made from WDR neurons in the spinal cord as described previously [29,30,31]. Mice were anesthetized with 2% isoflurane. A laminectomy was performed at vertebral levels T12 to L1 to expose the spinal lumbar enlargement with the dura mater incised and retracted. The L4–5 spinal segments were bathed in a pool of warm ACSF (35–37 °C), and other exposed areas were covered with warm agar (1.5%). The L4 DRG was also exposed, the sheath covering the surface of the DRG (perineurium and epineurium) was removed, and DRG was bathed in a pool of warm ACSF.

As in the calcium imaging experiments, drug solution was applied to the bath to the final working concentration for ganglionic drug treatment. A core body temperature (36.0–37.0 °C) was maintained by placing the mouse on a pad of circulating hot water. Extracellular activities of WDR neurons were recorded with fine-tip (<1.0 μm) paralyn-coated tungsten microelectrodes (1–3 mΩ at 1 kHz; Frederick Haer Company, Bowdoin, ME, USA). Signals were amplified and filtered (high pass: 300 Hz, low pass: 10 kHz; model DAM80; World Precision Instruments Inc., Sarasota, FL, USA). Analog data were collected in real-time with a computer-based data acquisition and processing system (CED Spike 2, Cambridge, UK). We quantified the number of action potentials evoked by electrical stimulation applied to the sciatic nerve. WDR cells were identified according to their characteristic responses [29].

### 2.5. Immunofluorescence

Mice were deeply anesthetized with pentobarbital and perfused intracardially with 1× PBS followed by 4% paraformaldehyde. L4–L5 DRG were dissected out and then post-fixed in 30% sucrose solution for 24 h for cryoprotection. Ten-micrometer sections were cut for DRG with a cryostat (CM3050s, Leica, Wetzlar, Germany). Sections were blocked in 5% normal goat serum and 1% Triton X-100 in PBS for 1 h at room temperature. After blocking, tissue sections were incubated overnight with primary antibodies against P2X3 (AB5896, Millipore; 1:1000), GFP (GFP-1020, Aves labs; 1:500), and GFAP (MAB360, Millipore; 1:500). The slides were incubated with the appropriate secondary antibodies including Alexa 488-conjugated goat antibody to chicken (A-11 039; Thermo Fisher Scientific, Waltham, MA, USA), Alexa 568-conjugated goat antibody to rabbit (A-11036; Thermo Fisher Scientific, Waltham, MA, USA), and Alexa 350-conjugated goat antibody to rabbit (A-11045; Thermo Fisher Scientific, Waltham, MA, USA) for 1 h at room temperature and then photographed using a confocal imaging system (LSM 700; Zeiss, White Plains, NY, USA). Raw confocal (TIFF) images (LSM 700; Zeiss, White Plains, NY, USA) were analyzed with Fiji (NIH) [32]. Two sections from each DRG were chosen for quantitative analysis (2 DRGs/mice, *n* = 3 mice). Positively stained neurons had clear somata and fluorescence intensity ≥ 30% of the background. For double-label and triple-label colocalization, images from the same DRG sections but showing different antigen signals were overlaid through digitally merging.

### 2.6. Drugs

BzATP, P2X7R antagonist A438079 hydrochloride hydrate, P2X3R antagonist A317491 sodium salt hydrate, and probenecid were purchased from Sigma-Aldrich (St. Louis, MO, USA). Other drugs were purchased from Sigma-Aldrich or Tocris Bioscience (Bristol, UK). Stock solutions were freshly prepared as instructed by the manufacturer.

### 2.7. Statistical Analysis 

Data were analyzed with the Prism 8.0 statistical program (GraphPad Software, Inc., San Diego, CA, USA). The methods for statistical comparisons in each study are given in the figure legends. Data that followed a normal distribution are expressed as mean ± SEM. We randomized animals to the different treatment groups and blinded the experimenter to drug treatment to reduce selection and observation bias. Two-tailed tests were performed, and *p* < 0.05 was considered statistically significant in all tests.

## 3. Results

### 3.1. BzATP Induced a Concentration-Dependent Activation of SGCs

**Figure 1:**
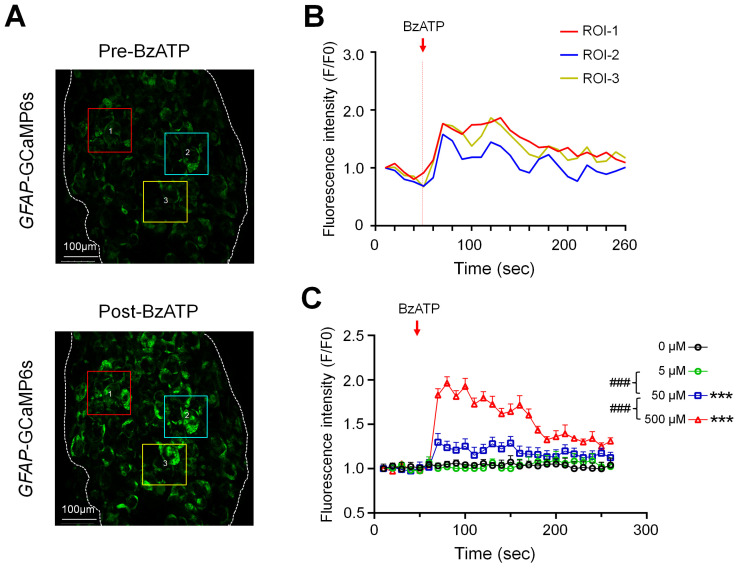
Concentration-dependent responses of satellite glial cells (SGCs) to the ganglionic application of BzATP. (**A**) Representative images illustrate the fluorescence intensity of SGCs at baseline (Frames (F)1–5, 10 s/frame) and 0–20 s (F6–7) after ganglionic application of BzATP (500 µM) in *GFAP*-GCaMP6s mice. For illustrative purposes, three regions of interest (ROIs) are marked with colored boxes and numbered. (**B**) Fluorescence intensity traces (F/F_0_) of the ROIs shown in (**A**) before and after the BzATP (500 µM) treatment. Fluorescence intensity was measured for each frame (10 s/frame) and plotted against time or frame number. (**C**) SGC responses in *GFAP*-GCaMP6s mice were quantified by measuring the averaged fluorescence intensity in the whole imaging field before (F_0_) and after (F) drug treatment. Fluorescence intensity was measured for each frame and plotted against time after ganglionic application of BzATP (5, 50, 500 µM, *n* = 6 mice/dose). The red arrow indicates the time of drug application. Data are expressed as mean ± SEM. *** *p* < 0.001 versus vehicle (0 µM); ### *p* < 0.001 versus indicated group. Two-way repeated measures ANOVA.

Ganglionic application of BzATP (5, 50, 500 µM, *n* = 6 mice/dose), which preferentially activates P2X7Rs, evoked prolonged responses of SGCs in *GFAP*-GCaMP6s mice in a concentration-dependent manner (Figure 1). Examples of SGC responses in selected regions of interest (ROI) are presented as images and traces of calcium transients (Figure 1A,B, Supplemental Figure S1A,B). Due to their irregular shapes, the size and response of individual SGCs were difficult to measure. To avoid bias in selecting ROIs, we measured the averaged fluorescence intensity in the whole imaging field to quantify the population SGC responses for each frame and then plotted it against time (Figure 1C). Time course analysis showed a prolonged excitation of SGCs in response to 500 µM BzATP. The increase in fluorescence intensity after BzATP application, i.e., area under the curve from Frames (F) 6–26, lasted over 100 s (>10 frames, Figure 1C).

### 3.2. More Large Neurons Than Small Neurons Were Activated by BzATP

**Figure 2:**
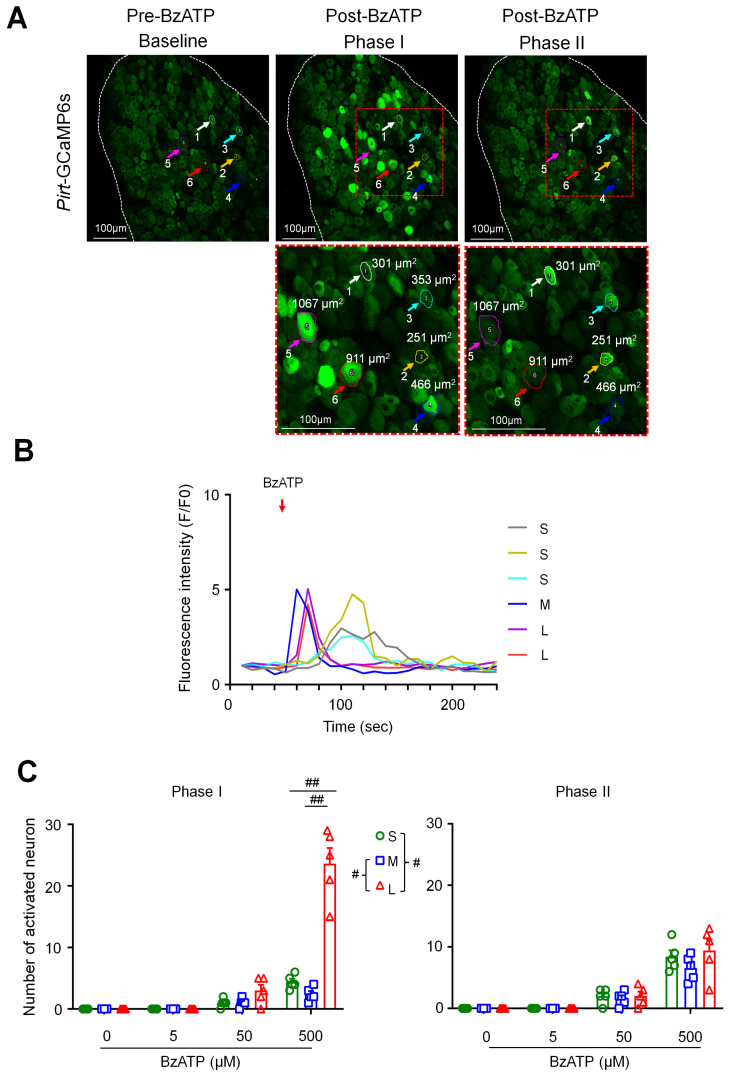
BzATP activates more large than small dorsal root ganglion (DRG) neurons in phase I. (**A**) *Upper*: Representative images showing changes in fluorescence intensity of L4 DRG neurons after ganglionic administration of BzATP (500 µM) in *Pirt*-GCaMP6s mice during in vivo calcium imaging in anesthetized mice. Baseline (Frames (F)1–5, 10 s/frame), phase I (F6–7), and phase II post-drug (F8–26). Examples of activated neurons are marked by colored circles and arrows. *Lower*: The red-outlined areas in phase I and phase II image are shown at higher magnification. DRG neurons were categorized according to the somal area as <450 μm^2^ (small, S), 450–700 μm^2^ (medium, M), and >700 μm^2^ (large, L). (**B**) Fluorescence intensity traces (F/F_0_) of selected neurons activated by BzATP in (**A**). Red arrow indicates the time of drug application. (**C**) Number of neurons in each subpopulation that were activated by BzATP (5, 50, 500 µM, *n* = 6 mice) in phase I (F6–7) and phase II (F8–26). Data are expressed as mean ± SEM. # *p* < 0.05, ## *p* < 0.01 versus indicated group. Two-way mixed-model ANOVA with Dunnett’s multiple comparisons test.

We then examined the responses of DRG neurons to ganglionic application of BzATP (5, 50, 500 µM, *n* = 6 mice) in *Pirt*-GCaMP6s. DRG neurons can be separated into three subpopulations with soma areas of <450 μm^2^ (small), 450–700 μm^2^ (medium), and >700 μm^2^ (large). Neuronal responses evoked by BzATP were usually transient (<1 min, Figure 2A,B) and are presented as raw images (Figure 2A) and traces of changes in cytosolic fluorescence intensity (Figure 2B). BzATP increased the number of activated neurons in a concentration-dependent manner (Figure 2C). Based on the observed response patterns, we noticed two phases of neuronal activation by BzATP: more large-size neurons showed a robust and earlier activation (within 20 s post-drug) as compared to small-size neurons. Accordingly, we separated the number of activated neurons into these two phases. At 500 µM, BzATP activated more large neurons than small and medium neurons in the early phase I (F6–7, 0–20 s post-drug; Figure 2C). In phase II (F8–26), the activation of large neurons decreased, and the number of neurons in each subpopulation was comparable (Figure 2C).

### 3.3. A317491, A438079, or Probenecid Inhibited the Excitation of Neurons and SGCs by BzATP

**Figure 3:**
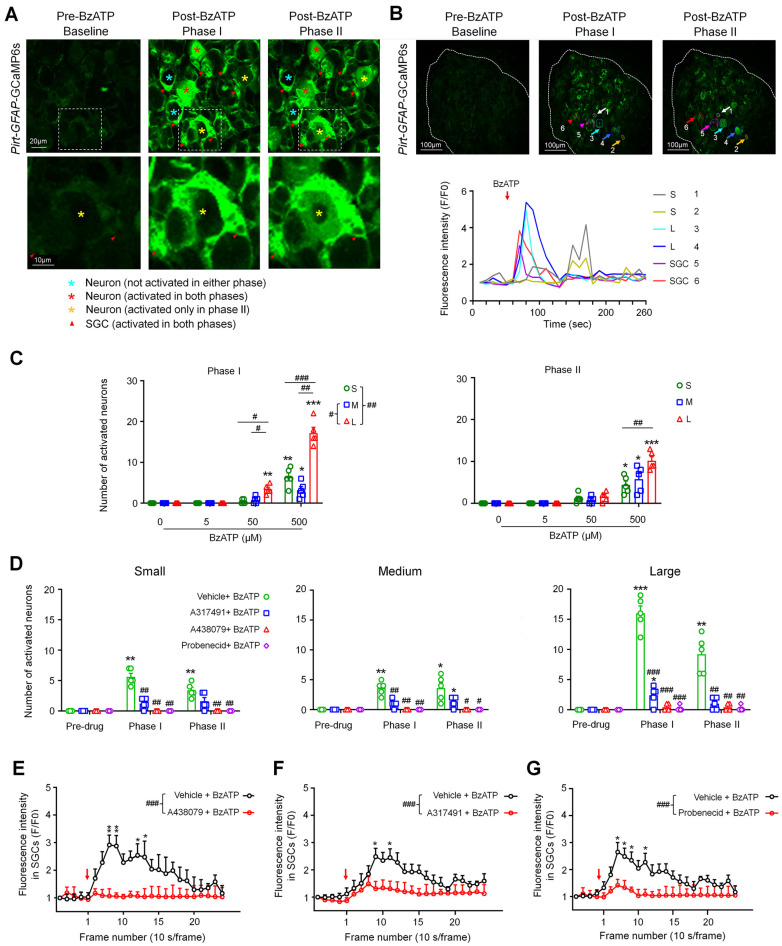
Pretreatment with A317491, A438079, and probenecid inhibited the activation of neurons and satellite glial cells (SGCs) by BzATP. (**A**) *Upper*: Representative images showing DRG neurons and SGCs after ganglionic administration of BzATP (100 µM) in *Pirt:GFAP*-GCaMP6s mice. Baseline (Frames (F)1–5, 10 s/frame), phase I (F6–7), and phase II post-drug (F8–26). *Lower*: The high-magnification images of the boxes outlined in the upper panel. (**B**) *Upper*: Examples of activated neurons (#1–4) and SGCs (#5–6) are marked with colored circles and arrows. DRG neurons were categorized by somal area as <450 μm^2^ (small, S), 450–700 μm^2^ (medium, M), and >700 μm^2^ (large, L). *Lower*: Fluorescence-intensity traces (F/F_0_) of DRG neurons and SGCs activated by BzATP. (**C**) Number of neurons in each subpopulation activated by BzATP (5, 50, 500 µM, *n* = 6 mice) in phase I and phase II. Data are expressed as mean ± SEM. * *p* < 0.05, ** *p* < 0.01, *** *p* < 0.001 versus vehicle (0 µM); # *p* < 0.05, ## *p* < 0.01, ### *p* < 0.001 versus indicated group. Two-way mixed-model ANOVA with Dunnett’s multiple comparisons test. (**D**) Number of neurons in each subpopulation that were activated by BzATP (100 µM) after pretreatment with vehicle (ACSF), the P2X3R antagonist A317491 (100 µM), the P2X7R antagonist A438079 (100 µM), or the Panx1 antagonist probenecid (1 mM; *n* = 6 mice/group). Data are expressed as mean ± SEM. * *p* < 0.05, ** *p* < 0.01, *** *p* < 0.001 versus pre-drug; # *p* < 0.05, ## *p* < 0.01 versus vehicle + BzATP in the same phase. Two-way mixed-model ANOVA with Tukey’s multiple comparisons test. (**E**–**G**) Average SGC fluorescence intensity produced by BzATP (100 µM) application after pretreatment with A438079 (100 µM, *n* = 6 mice, **E**), A317491 (100 µM, *n* = 5 mice, **F**), probenecid (1 mM, *n* = 6 mice, **G**), or vehicle (ACSF, *n* = 6 mice). Fluorescence was measured for each frame and then plotted against time. Red arrow indicates the time of drug application. The curves representing the changes in fluorescence intensity after different drug treatments were compared between groups. Data are expressed as mean ± SEM. * *p* < 0.05, ** *p* < 0.01 versus antagonist + BzATP; ### *p* < 0.001. Two-way mixed-model ANOVA with Tukey’s multiple comparisons test.

In *Pirt:GFAP*-GCaMP6s mice, responses of DRG neurons and SGCs can be monitored simultaneously (Figure 3A,B). Similarly to that in *Pirt*-GCaMP6s mice, BzATP (5, 50, 500 µM, *n* = 6 mice) concentration-dependently induced more activation of large neurons than small neurons in phase I in *Pirt:GFAP*-GCaMP6s mice (Figure 3B,C). To avoid the ceiling effect, we chose a moderate concentration (100 µM) of BzATP based on a concentration–response study to examine its receptor mechanisms for the activation of neurons and SGCs. We pretreated the DRG with the P2X3R antagonist A317491 (100 µM), the P2X7R antagonist A438079 (100 µM), probenecid (a pannexin1 blocker; 1 mM), or vehicle at 2–3 min before adding BzATP (100 µM, *n* = 6 mice/group). The concentration of each antagonist was based on previous studies [6,10,33,34]. Different antagonists were tested in different animals. Pretreatment with any of the three drugs significantly reduced the neuronal activation by BzATP in both phases (Figure 3D, *n* = 6 mice/group) as compared to vehicle. Notably, a pretreatment with A438079 (100 µM, *n* = 6 mice, Figure 3E, Supplemental Figure S1C), A317491 (100 µM, *n* = 5 mice, Figure 3F), or probenecid (1 mM, *n* = 6 mice, Figure 3G) also inhibited the responses of SGCs to BzATP.

### 3.4. Ganglionic Application of BzATP Sensitized DRG Neurons

**Figure 4:**
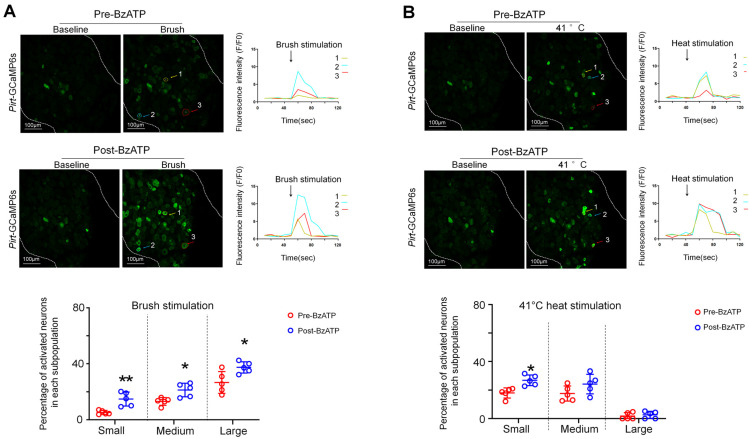
Changes in the percentage of DRG neurons that respond to brush and heat stimulation after ganglionic application of BzATP. (**A**) *Upper*: Representative images show L4 DRG neuronal fluorescence in response to brush stimulation (~1 Hz for 10 s) at the hind paw before and 5 min after BzATP (100 µM) application in *Pirt*-GCaMP6s mice. Fluorescence-intensity traces (F/F_0_) of the activated neurons marked with colored arrows. *Lower*: Percentage of neurons in each size of population that responded to brush stimulation before and after BzATP treatment (*n* = 5 mice). DRG neurons were categorized according to somal area as <450 μm^2^ (small), 450–700 μm^2^ (medium), and >700 μm^2^ (large). (**B**) *Upper*: Representative images show neuronal fluorescence in response to non-noxious heat stimulation of the hind paw (41 °C water bath, 10 s) before and 10 min after BzATP (100 µM) application. *Lower*: Percentage of neurons in each subpopulation that responded to heat stimulation (*n* = 5 mice). Data are expressed as mean ± SEM. * *p* < 0.05, ** *p* < 0.01 versus pre-drug. Paired *t*-test.

We further examined how a brief treatment with BzATP might alter neuronal responses to subsequent afferent inputs. Brush stimulation alone (~1 Hz, 10 s) at the ipsilateral hind paw increased fluorescence intensity primarily in large neurons (Figure 4A), whereas heat stimulation (41 °C water bath, 10 s) activated mostly small and medium neurons (Figure 4B). At 5 min after BzATP (100 µM) treatment, when the neuronal responses to BzATP had diminished and returned to baseline, the percentage of neurons in each subpopulation that responded to brush stimulation significantly increased from the pre-drug level (Figure 4A, *n* = 5 mice). Upon heat stimulation, this treatment increased only the percentages of responding small neurons (Figure 4B, *n* = 5 mice).

Carbenoxolone (CBX) is a gap junction decoupler and can induce pain inhibition in a variety of animal models [6,34,35,36,37]. Peripheral noxious stimulation may induce the release of ATP in the DRG and activate neurons and SGCs. In the current study, the ganglionic application of CBX (100 µM) decreased the number of DRG neurons activated by peripheral high-intensity electrical stimulation (*n* = 5 mice, Supplemental Figure S2)

### 3.5. Spinal WDR Neuron Responses to Electrical Stimulation Were Increased after Ganglionic Application of BzATP

**Figure 5:**
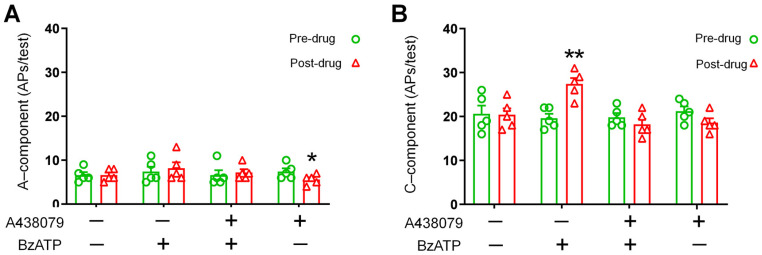
Effects of ganglionic BzATP application on spinal wide-dynamic-range (WDR) neuronal responses to electrical stimulation. (**A**) WDR neuron responses to supra-threshold electrical stimulation (3.0 mA, 2 ms) at the sciatic nerve were separated into A- and C-fiber components (number of action potentials) based on latency. Quantification of A-component and (**B**) C-component response to electrical stimulation before and 5 min after ganglionic application of BzATP (100 µM) with and without the P2X7R antagonist A438079 (100 µM, *n* = 5 mice/group; 1 mM, 200 µL), which was applied to a bath of ~2 mL ACSF 2 min before the agonist was added. Data are expressed as mean ± SEM. * *p* < 0.05, ** *p* < 0.01 versus pre-drug. Paired *t*-test.

WDR neurons from the deep dorsal horn (350–700 µm below the surface) receive both A-fiber and C-fiber inputs and play an important role in spinal nociceptive transmission [29,30,38]. Based on the activation thresholds and latencies, the responses of WDR neurons to high-intensity electrical stimulation (3.0 mA, 2 ms) at the sciatic nerve were separated into A-fiber- and C-fiber-mediated components, as shown in our previous studies [29,30]. At 5–10 min after application of BzATP (100 µM) to L4 DRG, the A-component responses of WDR neurons to the electrical stimulation were not changed (Figure 5A). However, the C-component was significantly increased (Figure 5B), as compared with the pre-drug level. This effect was blocked by pretreatment with DRG application of A438079 (100 µM, *n* = 5 mice/group) 2 min before adding BzATP. These results suggest that the excitability of DRG neuronal somata was enhanced after ganglionic BzATP treatment and increased transmission of nociceptive inputs from the periphery to the spinal cord.

### 3.6. Expression of P2X3 Receptors in DRG of Pirt-GCaMP6s Mice

**Figure 6:**
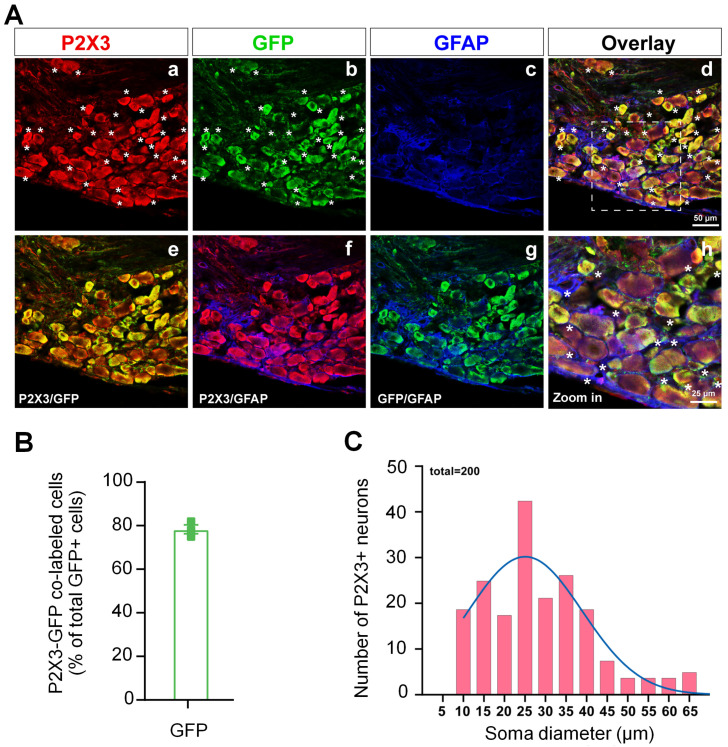
Expression of P2X3 protein in the dorsal root ganglion (DRG) of *Pirt*-GCaMP6s mice. (**A**) The P2X3 receptor immunoreactivity was detected in neurons, but not satellite glial cells (SGCs), in the DRG of *Pirt*-GCaMP6s mice. The distribution of P2X3 (red), GFP (green, which labels GCaMP6s-expressing neurons), and GFAP (blue, which labels SGCs) fluorescence signals in the DRG. * indicates P2X3 and GFP co-labeled neurons. (a,b,c) Single-label images of P2X3, GFP and GFAP. (d) An overlaid image of (a–c). (e,f,g) Double-label images. (h) A high-magnification image of the box outlined in (d). (**B**) Quantification of the percentage of P2X3–GFP co-labeled neurons in total GFP-positive neurons (*n* = 3 mice). (**C**) Distribution of somata diameters (5 µm bins) of P2X3-positive (+) neurons from lumbar DRGs of *Pirt*-GCaMP6s mouse (*n* = 200 neurons from 3 mice).

Our triple-labeling study showed that the immunoreactivity of P2X3R was present mostly in the neurons in the DRGs from *Pirt*-GCaMP6s mouse. *Pirt* is selectively expressed in >85% of mouse DRG neurons but not in the SGCs. Since the expression of GCaMP6s is driven by the *Pirt* promotor in *Pirt*-GCaMP6s mice, DRG neurons expressing GCaMP6s can be readily stained with GFP antibody (green). Our findings show that P2X3R (red) is co-localized with GFP in a large portion of GFP+ neurons (78.33%, Figure 6A,B), but does not co-localize with GFAP (blue) which labels SGCs [39,40]. Accordingly, the P2X3R is mostly, if not exclusively, expressed in the neurons in mouse DRG. The size distribution analysis shows that P2X3R+ neurons are mainly small- (<30 μm) to medium-diameter cells (30–50 μm, Figure 6C).

## 4. Discussion

Using in vivo GCaMP6s imaging of DRG, we found that the ganglionic application of BzATP induced concentration-dependent activation of both SGCs and neurons. Moreover, BzATP increased DRG neuron excitability and facilitated the transmission of nociceptive signals to the spinal cord. These results extend our recent findings of α,β-MeATP-evoked ganglionic purinergic signaling, which similarly increased DRG neuron excitability [10].

Unlike α,β-MeATP, which preferentially activates neuronal P2X3R, BzATP can activate P2X1R, P2X4R, and P2X7R [5,21,22]. Since P2X1R is minimally expressed in DRG, BzATP likely acts on DRG cells via P2X4R and P2X7R. P2X7R is abundant in SGCs, implying that BzATP is a potent SGC activator. Two previous studies indicated that some medium and large DRG neurons in cat and guinea pig may express P2X7R [41,42]. However, this finding remains to be confirmed in mouse DRG. The picture is also less clear for P2X4R. Earlier studies indicated that DRG neurons express P2X4R, with even distribution in all neuron sizes [43,44,45]. However, Chen et al. (2016) showed that immunoreactivity for both P2X4R and P2X7R in DRG neurons is much lower than that of P2X3R [46], suggesting that they may only play a minor role in neuronal purinergic signaling under basal conditions. Moreover, a recent study demonstrated that P2X4R in DRG was present only in SGCs, but not in neurons [47]. Although an *a priori* suggestion that BzATP will selectively stimulate SGCs via P2X7R may seem uncertain, our observation that BzATP-induced SGC and neuronal activation were completely blocked by the P2X7R blocker A438079 supports this assumption.

Previous studies suggested that SGCs exerted an inhibitory action on neuronal activity under normal physiological conditions [48,49]. However, our in vivo study showed that BzATP, which strongly activates SGCs, also induced neuronal calcium responses. Further analysis revealed that BzATP activated a greater number of large neurons than small neurons in the early phase. In addition to P2X7R, at a high concentration, BzATP may have also activated the aforementioned P2X4R. Accordingly, a high concentration of BzATP may directly excite DRG neurons. However, our findings showed that the P2X7R antagonist A438079 completely inhibited BzATP-induced neuronal excitation, suggesting that this effect may be a consequence of the activation of P2X7R on SGCs, possibly by ATP that was released from SGCs and activated neuronal P2Rs.

**Figure 7:**
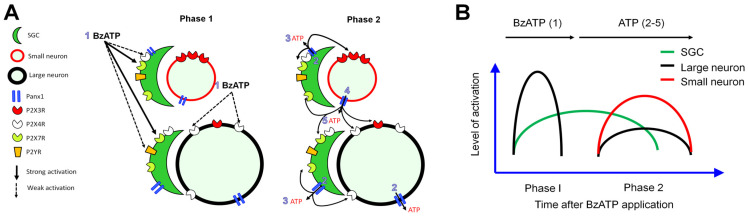
Hypothetical model illustrating the activation of DRG neurons and SGCs by BzATP. (**A**) 1. In phase I, BzATP strongly activates P2X7Rs, which are abundant in SGCs. To a smaller degree, BzATP may also activate other P2XR (e.g., P2X4R), which may be expressed on SGCs and large DRG neurons, as suggested by previous findings, and P2YR on SGCs. 2. In phase 2, the activation of SGCs and large neurons leads to Panx1 opening and release of ATP. 3. ATP further activates both small and large neurons and SGCs. 4. The activation of small neurons also leads to Panx1 opening and release of ATP to the extracellular space. 5. ATP, in turn, activates more SGCs and neurons. This process ends rapidly as ATP is degraded or cleared from the extracellular space. Neurons and SGCs return to the resting state, but neurons may become sensitized to subsequent stimulation. (**B**) The diagram illustrates the sequential activation of large neurons, SGCs, and small neurons after BzATP treatment.

The activation of P2X7R is essential to opening the downstream Panx1 channels in glial cells [6,50], and ATP release is a key consequence of Panx1 channel opening [1,50,51]. Panx1 may play an important role in pain by controlling the ATP release [1,6,51]. In nodose ganglia, Panx1 was thought to be present only in neurons [52]. However, there is strong recent evidence for the presence of Panx1 in both neurons and SGCs in nodose ganglia [53], and in trigeminal ganglia of adult animals [54,55]. Our findings that probenecid attenuated BzATP-induced neuronal activation imply the involvement of downstream Panx1 channels in this process. Collectively, these findings suggest that purinergic signaling initiated by BzATP in SGCs may elicit interactions with neurons in vivo. One scenario is that the activated SGCs release ATP [4,18,48], which in turn activated neurons through other purinergic receptors. A317491 attenuated BzATP-induced neuronal activation, suggesting that the activation of neuronal P2X3Rs was also involved. Thus, there may be a cascade of early cellular activation by BzATP, followed by secondary paracrine action of endogenous ATP (Figure 7). This may partially explain the involvement of different P2X receptors and Panx1 channels in the observed drug actions. Nevertheless, because pharmacological studies are often limited by potential non-selective drug actions, the detailed mechanisms remain to be examined in future studies. For example, it is unexpected that probenecid also inhibited the excitation of SGCs to BzATP, as BzATP can directly activate SGCs through P2X7Rs and does not depend on Panx1 channels. We postulate that the inhibition of SGCs by probenecid may be partially due to its Panx1-independent actions. In addition to blocking Panx1 channels, probenecid may also directly impair canine P2X7R activation and inhibit the transport of organic anions through P2X7Rs [56,57,58].

The activation of P2X7R may trigger the glial release of ATP and proinflammatory mediators, which can induce neuronal sensitization in vitro [4,48]. For example, ATP activates P2X7R on SGCs, which subsequently release TNFα to sensitize P2X3R in cultured neurons [4]. However, ATP may also directly activate neuronal P2XR and sensitize neurons. Importantly, whether the activation of P2X7R on SGCs can affect neuronal excitability and nociceptive transmission in DRG in vivo remains unclear. Here, using a potent, P2X7R-preferential agonist BzATP, we confirmed previous in vitro findings and provided novel evidence that the ganglionic application of BzATP not only activated SGCs but also increased calcium responses of small-size DRG neurons to subsequent non-noxious mechanical and heat stimulation at the hind paw. Moreover, C-fiber-mediated responses of spinal cord WDR neurons to electrical stimulation were also increased after the ganglionic application of BzATP in vivo. These effects of BzATP were blocked by applying the P2X7R antagonist A438079 on the ganglion, supporting previous in vitro observations that the activation of P2X7R on SGCs is important to DRG neuronal sensitization [1,3,10,17,59,60].

SGCs may also participate in calcium waves that spread to neurons via gap junctions and P2Rs [3,4]. The ganglionic application of CBX decreased the number of DRG neurons activated by peripheral high-intensity electrical stimulation, suggesting that the inhibition of gap junctions in the sensory ganglion may attenuate neuronal responses to peripheral afferent inputs. However, it needs to be noted that CBX is nonselective. CBX also blocks pannexin1 channels and could have effects on ion channels that also affect neuronal excitability [17,18]. GCaMP6s is highly sensitive to changes in intracellular calcium level, and in vivo calcium imaging can be used to survey a large number of neurons and SGCs and allow unequivocal characterization of their activities in intact mice [10,61,62,63]. However, calcium imaging has limitations, mainly slow temporal kinetics and a long recovery from the fluorescent state [25,62]. Additionally, in contrast to electrical recordings, fast and transient changes in neuronal activities may not be detected.

## 5. Conclusions 

In summary, the current findings extended our recent observations that in vivo ganglionic application of α,β-MeATP elicited robust neuronal and SGC responses, and increased neuronal excitability [10]. Due to potential off-target effects of drug treatment, the target specificity and cellular mechanisms of the aforementioned in vivo drug actions on different cell types warrant further investigation, using transgenics (e.g., P2X3R and P2X7R knockout mice), siRNA treatment, chemogenetics, or optogenetics to induce cell-type-specific modulation. Nevertheless, these findings further demonstrate the important roles of intra-ganglionic purinergic signaling in modulating neuronal excitability and nociceptive transmission, beyond its function in sensory transduction at the nerve terminals. Since DRGs are not protected by a blood–nerve barrier because the endothelium of blood vessels that supply DRG lacks tight junctions [64,65], developing peripherally acting drugs (i.e., not crossing the blood–brain barrier after systemic administration) to target purinergic receptors on SGCs (e.g., P2X7R) and neuronal somata (e.g., P2X3R) in DRG may be a new strategy for pain treatment to avoid potential central side effects.

## Data Availability

Not applicable.

## Supplementary Materials

The following supporting information can be downloaded at: https://www.mdpi.com/article/10.3390/cells11152280/s1, Figure S1: Activation of satellite glial cells (SGCs) to the ganglionic application of BzATP was blocked by P2X7 receptor antagonist pretreatment. Figure S2: Ganglionic application of Carbenoxolone (CBX) decreased the number of DRG neurons responding to peripheral high-intensity electrical stimulation.
