# Supplementary material for: BzATP Activates Satellite Glial Cells and Increases the Excitability of Dorsal Root Ganglia Neurons In Vivo
Source: Cells. 2022 Jul 23;11(15):2280. doi: 10.3390/cells11152280 (PMC9330736; doi:10.3390/cells11152280)
Supplement: Supplementary file 1 [file cells-11-02280-s001.zip › cells-1731141-supplementary.pdf]

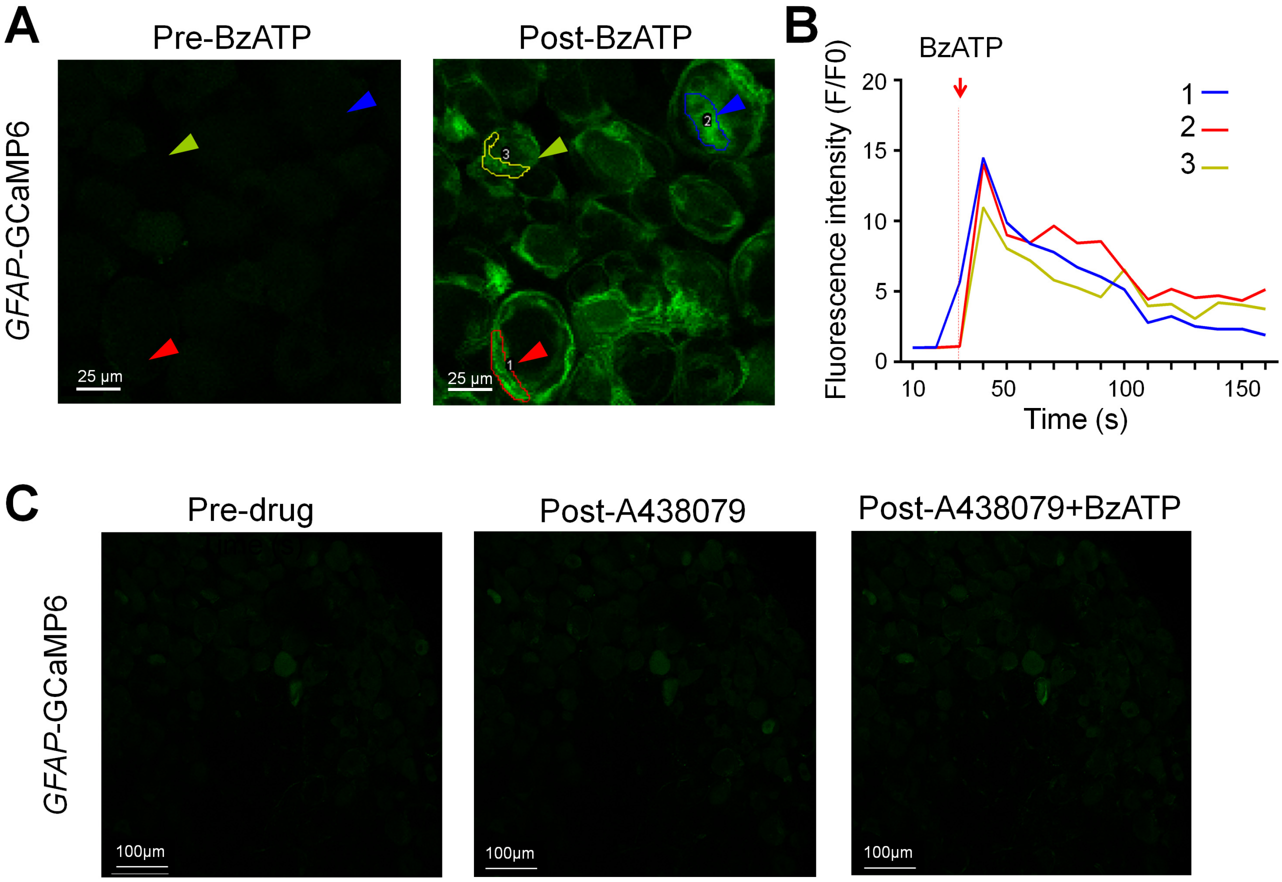

Figure S1. Activation of satellite glial cells (SGCs) to the ganglionic application of BzATP was blocked by P2X7 receptor antagonist pretreatment. (**A**) Representative images illustrate the fluorescence intensity of SGCs at baseline [Frames (F)1-5, 10 sec/frame] at 0-20 sec (F6-7) after ganglionic application of BzATP (100 μM) in *GFAP*-GCaMP6s mice. For illustrative purposes, three SGCs are marked with colored boxes and numbered. (**B**) Fluorescence intensity traces (F/F_0) of the three SGCs shown in (A) before and after the BzATP (100 μM) treatment. Fluorescence intensity was measured for each frame and plotted against time. (**C**) Representative images of calcium transient in SGCs at baseline [Frames (F)1-5, 10 sec/frame], 0-20 sec (F6-7) after ganglionic application of A438079 (100 μM, a selective P2X7 receptor antagonist), and at 0-20 sec after BzATP (100 μM) treatment. BzATP was applied at 3 min after A438079 pre-treatment.

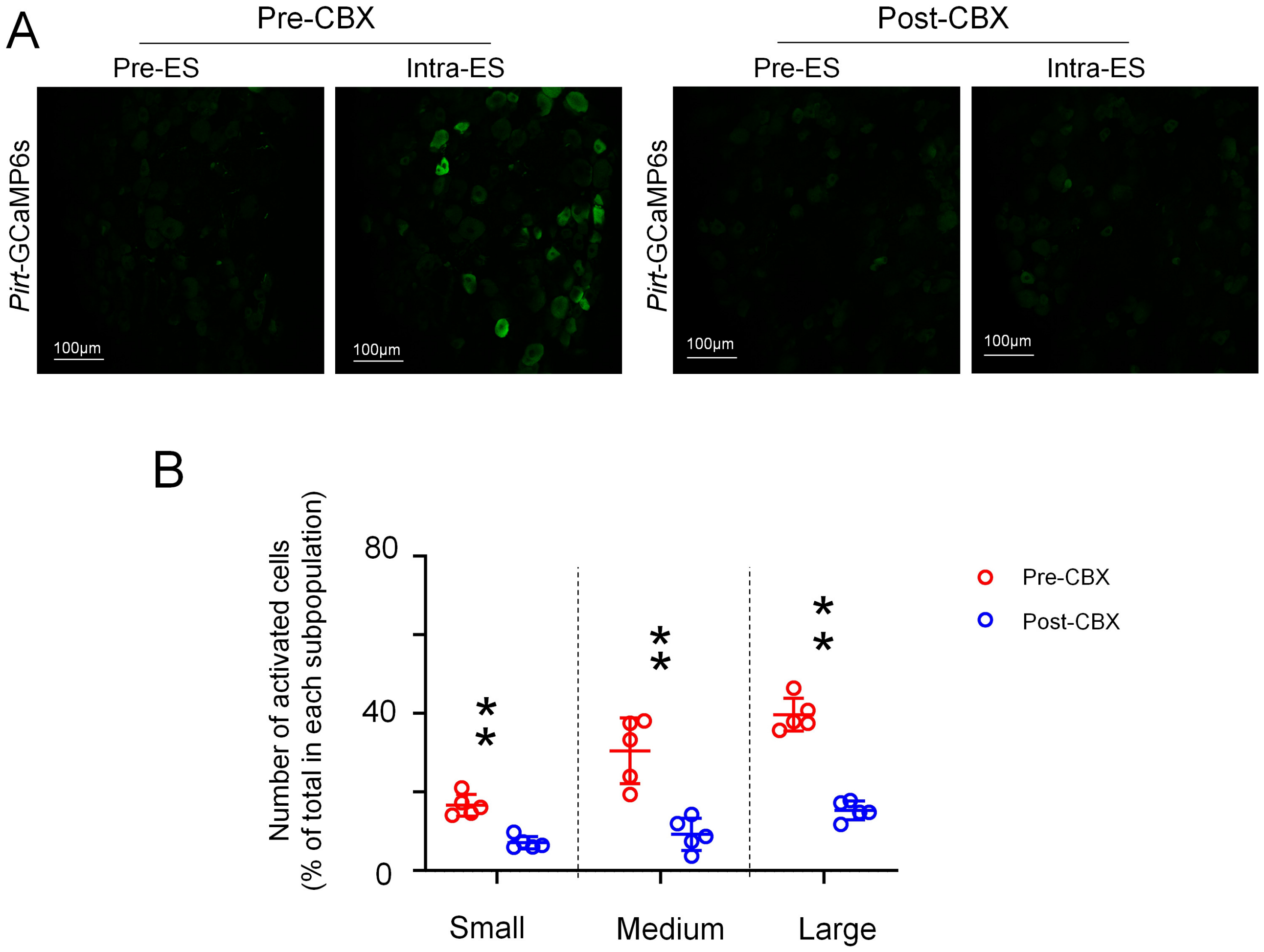

FigureS2. Ganglionic application of Carbenoxolone (CBX) decreased the number of DRG neurons responding to peripheral high-intensity electrical stimulation. (**A**) Representative images of L4 DRG neurons to the electrical stimulation (ES, 3.0 mA, supra-threshold to small neuron activation, 2 ms, 2 Hz, 8 s) at the hind paw before (left) and 5 min after (right) ganglionic application of CBX (100 μM) in *Pirt*-GCaMP6s. (**B**) Quantification of the number of neurons in each subpopulation responding to the same electrical stimulation before and after CBX treatment (n = 5 mice). DRG neurons were categorized into three sized-based subpopulations with somal areas of <450 μm^2 (small), 450–700 μm^2 (medium), and >700 μm^2 (large). Data are expressed as mean ± SEM. **P < 0.01 versus pre-drug. Paired t-test.
